# Case Report: Pediatric ACTH-secreting pituitary adenoma presenting with hypertension and anuria

**DOI:** 10.3389/fendo.2026.1894223

**Published:** 2026-07-08

**Authors:** Jiaxin Li, Wei Xia, Mengxing Wu

**Affiliations:** 1 School of Medicine, Jianghan University, Wuhan, China; 2 Wuhan Children's Hospital (Wuhan Maternal and Child Healthcare Hospital), Tongji Medical College, Huazhong University of Science and Technology, Wuhan, China; 3 Wuhan University of Science and Technology School of Medicine, Wuhan, China

**Keywords:** ACTH-Secreting pituitary adenomas, anuria, hypertensive, MRI, pituitary apoplexy

## Abstract

**Background:**

Adrenocorticotropic hormone (ACTH)-secreting pituitary adenomas are rare endocrine neoplasms. Pituitary apoplexy, caused by acute hemorrhage or infarction within the tumor, is an uncommon complication. Pediatric cases of ACTH-secreting adenomas with apoplexy are exceptionally rare, with limited understanding of their clinical and imaging manifestations. This report aims to enhance diagnostic awareness through a detailed case analysis.

**Case presentation:**

A 13-year-old boy presented with acute-onset severe headache, anuria, hypertensive crisis (180/120 mmHg), and rapid weight gain (20 kg over 2 months). Biochemical evaluation revealed elevated ACTH (190 pg/mL), disrupted cortisol rhythm, and hypokalemia (2.16–3.0 mmol/L). Pituitary magnetic resonance imaging (MRI) demonstrated a 6mm×5 mm cystic lesion in the left adenohypophysis with mixed T1/T2 signals and no post-contrast enhancement, consistent with apoplexy. Histopathology confirmed an ACTH-secreting adenoma. Transsphenoidal resection and hormone replacement achieved stable remission at the 3-year follow-up.

**Conclusions:**

Pediatric ACTH-secreting pituitary apoplexy presents diagnostic challenges due to nonspecific symptoms and atypical imaging features. MRI remains pivotal for initial evaluation, though histopathology is confirmatory. Early surgical intervention and hormonal management are critical for favorable outcomes.

## Background

Pituitary adenomas derive from hormone-secreting cell lineages, with clinical phenotypes dictated by their cellular origins and associated hormone hypersecretion. For instance, prolactinomas typically present with galactorrhea and infertility, while corticotroph adenomas manifest as hypercortisolism leading to Cushing’s disease—each exhibiting hormone-specific clinical features. Pituitary adenomas rarely occur in pediatric populations, constituting only 5% of all cases (1). Their prevalence is significantly lower than in adults, and they show a female predominance. Pituitary apoplexy occurs in 2%-12% of cases, predominantly in macroadenomas (>10 mm) and non-functioning tumors, while remaining exceptionally rare in microadenomas (2). ACTH-secreting subtypes account for <0.01% of cases (3) and cause Cushing’s disease, a clinically challenging condition frequently misdiagnosed owing to diagnostic intricacies. Clinical manifestations vary widely, often mimicking other acute neurological or endocrine emergencies. Imaging findings, particularly on MRI, may lack specificity, necessitating correlation with histopathology. Pediatric pituitary apoplexy is rare, with current evidence-based data predominantly derived from large-scale adult studies. We describe a pediatric case with atypical presentation and imaging features, emphasizing diagnostic pitfalls.

## Case presentation

A 13-year-old boy presented to the emergency department with a 1-day history of low back pain and anuria, followed by progression to severe headache over half a day, marked by two episodes of convulsive syncope with loss of consciousness. He was born at term after an uncomplicated pregnancy and had achieved all developmental milestones appropriately, with no prior hospitalizations or significant illnesses. At presentation, his height was 165 cm and weight was 85.5 kg (BMI 31.4 kg/m²), reflecting a rapid gain of 20 kg over the preceding two months. There was no family history of endocrine disorders, and he had no known drug allergies or regular medications. The family initially attributed his weight gain to normal pubertal growth but sought emergency care when he developed severe headache and anuria. Physical examination revealed densely distributed truncal acne and abdominal striae, with blood pressure severely elevated at 180/120 mmHg. Endocrine evaluation revealed markedly elevated ACTH (190 pg/mL; reference 0–46 pg/mL) and disruption of the normal cortisol circadian rhythm. Late-night serum cortisol was 1019.16 nmol/L (reference < 50 nmol/L), 8 AM cortisol was 1630.9 nmol/L (reference 145.4–619.4 nmol/L), and 4 PM cortisol was 1172 nmol/L (reference 94.9–462.4 nmol/L). Recurrent hypokalemia was also observed, with serum potassium fluctuating between 2.16 and 3.0 mmol/L (reference 3.7–5.2 mmol/L). Persistent anuria ensued despite hydration therapy during hospitalization, with minimal sanguineous urinary output (<50 mL) following catheterization, creatinine 237.7μmol/l (≥ 26.5μmol/l), antihypertensive therapy resulted in blood pressure readings of 148–160/89–104 mmHg, with systolic measurements predominantly ranging from 150 to 158 mmHg. Computed tomography (CT) demonstrated bilateral adrenal thickening, MRI of the pituitary demonstrated a cystic lesion in the left adenohypophysis with T1-weighted hypointensity and T2-weighted hyperintensity, punctuated by patchy T1-hyperintense areas (**Figure 1A**), measuring approximately 6 mm×5mm. Post-contrast imaging showed no enhancement (**Figure 1B**). Postoperative histopathology confirmed an ACTH-positive adenoma with hemorrhagic necrosis (**Figure 2**).

**Figure 1:**
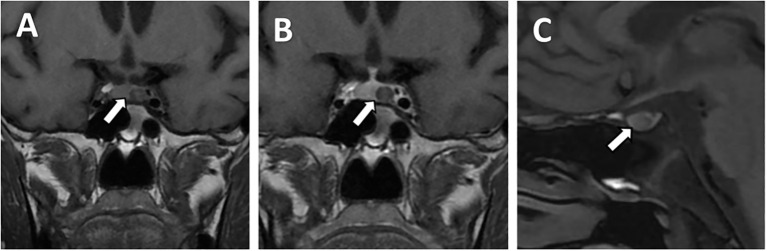
**(A)** T1-weighted noncontrast coronal images. The pituitary demonstrated a cystic lesion in the left adenohypophysis, with a patchy T1 high signal area suggestive of hemorrhage (arrow). **(B)** T1-weighted postcontrast coronal image demonstrates a pituitary adenoma with no enhancement (arrow) and no discernible mass effect on surrounding structures. **(C)** T1-weighted postcontrast sagittal image with a hyperenhancing normal pituitary gland (arrow).

**Figure 2:**
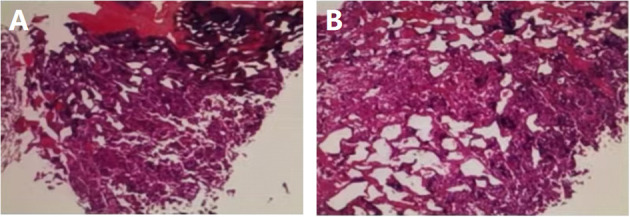
**(A, B)** Histological sections of the resected specimen. Gross appearance: gray-brown tissue, approximately 0.3×0.3×0.1 cm. Pathological diagnosis: (sellar lesion) pituitary adenoma. Immunohistochemistry: CK8/18 (+), Syn (+), ACTH (+), GH (+), PRL (+), LH (focal +), T-pit and Pit-1 (partial +), SF-1 and SOX-2 (focal +), FSH (-), TSH (-), P53 (focal weak +), Ki-67 proliferation index approximately 1%.

Transsphenoidal resection of the lesion was performed, followed by hormone replacement therapy (**Table 1**). Follow-up: At 3 years post-resection, the family were relieved to see that the child had normal blood pressure, weight, kidney function, and endocrine function, with no sign of recurrence.

**Table 1:** Timeline of key clinical events during hospitalization and follow-up.

Time period	Major clinical events/interventions	Laboratory/imaging findings	Fluid balance/catheter
Before admission	Low back pain, anuria; emergency ultrasound showed nephrolithiasis; antibiotics ineffective; later headache, convulsion, BP 180/120 mmHg	Ultrasound: bilateral nephrolithiasis, hydronephrosis, enlarged kidneys	—
Early hospitalization (days 1–4)	Hypertensive crisis, anuria, Cushingoid features; admitted to PICU; antihypertensives, mannitol, antibiotics	Elevated serum creatinine, BUN, uric acid; adrenal CT: bilateral thickening; abdominal CT: bilateral nephrolithiasis, bladder stones, infectious changes; brain MRI: possible hypertensive encephalopathy	Indwelling urinary catheter, initial anuria (<50 mL/24h); positive fluid balance (pericardial, pleural, pelvic effusions)
Renal recovery and preoperative evaluation	Renal function normalized; continued antihypertensive therapy; completed echocardiography, chest ultrasound, urine RBC morphology, renal ultrasound; started oral potassium citrate	Renal function normal; urine RBC morphology: isomorphic 72%, protein negative; renal ultrasound: enlarged kidneys, increased echogenicity, indistinct corticomedullary differentiation, pelvic separation	Urine output gradually recovered, catheter removed
Transsphenoidal pituitary tumor resection	Tumor resection under general anesthesia; intraoperative left cystic lesion with old bloody fluid; gross specimen: gray−brown tissue 0.3×0.3×0.1 cm	Pre−operative renal function and urinalysis normal	—
Early post−operative	Blood pressure decreased, some antihypertensives discontinued; pathology: ACTH−positive adenoma with hemorrhagic necrosis	Renal function and urinalysis remained normal	Fluid balance normalized, effusions resolved spontaneously
3−year follow−up	Complete clinical and biochemical remission, no recurrence	Renal function and urinalysis normal	—

## Discussion

ACTH-secreting adenomas account for 10%–12% of pituitary tumors (4). Apoplexy in such cases is rare, often triggered by tumor hemorrhage or infarction. Pituitary apoplexy lacks specific clinical manifestations and typically presents with sudden onset of headache with concurrent visual or endocrinological deterioration, secondary to abrupt sellar mass expansion from hemorrhage, infarction, or necrosis within the tumor and adjacent pituitary gland (4). The tumor in our patient measured 6mm, classifying it as a microadenoma. Pituitary apoplexy complicates 2%-12% of pituitary adenomas overall (2), but is exceptionally rare in microadenomas, with the vast majority of cases occurring in macroadenomas (5). Importantly, that review identified tumor size (macroadenoma) and non-functioning status as the only independent risk factors for apoplexy (5). Our case is therefore particularly noteworthy: it occurred in a microadenoma, and the tumor was functioning—both features that deviate completely from this typical risk profile. This raises the possibility that other factors—such as the degree of hormone hypersecretion or acute hemodynamic changes—may have contributed to the hemorrhagic event in this pediatric patient.

The diagnostic challenge and rarity of this presentation are further reflected in the literature. Zhu et al. reported that among 46 patients with suprasellar pituitary adenomas confirmed by surgery, preoperative imaging misidentified the tumor origin in 30.4% of cases (6), highlighting the inherent limitations of MRI in localizing sellar-suprasellar lesions. In the pediatric population, Li et al. found that among 232 children with pituitary neuroendocrine tumors, the majority were macroadenomas, and only one of 90 ACTH−secreting adenomas presented with cystic change or apoplexy (7), reinforcing the rarity of our presentation and the need for a high index of suspicion even when imaging findings are atypical.

Compared with previously reported cases, this case exhibited unique features in both clinical presentation and imaging findings. First, the patient developed hypertensive crisis-induced acute renal failure. This may be related to hypercortisolism-driven glomerular injury and renin-angiotensin system activation, which could contribute to progressive vascular injury and potentially lead to a self-perpetuating cycle of refractory hypertension. These mechanisms were not directly investigated in this case and should be considered speculative. Second, defying conventional renal pathophysiology, the patient showed paradoxical persistence of refractory hypokalemia despite acute renal failure—a finding possibly related to uncorrected cortisol excess driving ongoing potassium losses, compounded by impaired renal potassium conservation. This pathophysiological interpretation, while plausible, was not directly assessed in this patient. Third, imaging revealed an absence of classic MRI signs of apoplexy (e.g., peripheral nodular enhancement, sphenoid sinus mucosal thickening, or an air-fluid level); instead, a non-enhancing cystic lesion was seen, closely mimicking a Rathke’s cleft cyst.

In this case, imaging revealed a left-sided pituitary cyst with patchy T1-hyperintense signal suggesting hemorrhage; post-contrast sequences showed no enhancement. This finding raised the possibility of Rathke’s cleft cyst (RCC), which typically presents with headache, visual field defects, or hypopituitarism. However, the absence of contrast enhancement is not specific to RCC—in pituitary apoplexy, hemorrhagic infarction can also devascularize the adenoma, leading to no enhancement. As Zhu and colleagues noted, suprasellar cystic lesions without enhancement can closely mimic RCCs on MRI (6). The key differentiator in our patient was the internal T1-hyperintense signal (subacute hemorrhage) combined with frank ACTH-dependent hypercortisolism—features not seen in RCCs, which do not cause hormone hypersecretion (8). Thus, despite radiological overlap, the presence of hemorrhage and hormone excess firmly supports apoplexy over a primary cystic lesion.

Hypertensive encephalopathy was initially suspected based on severe hypertension (180/120 mmHg) and neurological symptoms (headache, convulsive syncope). However, this diagnosis was contradicted by the absence of typical MRI findings of posterior reversible encephalopathy syndrome (PRES) and the presence of ACTH-dependent hypercortisolism. Pheochromocytoma crisis was also considered. The patient lacked classic paroxysmal symptoms (palpitations, sweating, headache) and instead exhibited overt Cushingoid features. Moreover, although approximately 90% of pheochromocytomas arise from the adrenal medulla, the lesion in our child was located in the suprasellar region—a highly atypical site. The internal T1-hyperintense signal on MRI indicated subacute hemorrhage, consistent with pituitary apoplexy; this differs from the marked T2 hyperintensity (“light bulb sign”) characteristic of pheochromocytoma. Finally, sustained remission of hypertension after transsphenoidal resection of the ACTH-positive adenoma confirmed that the hypertensive crisis was due to ACTH-dependent hypercortisolism, not catecholamine excess. Collectively, these findings do not support pheochromocytoma. In our patient, overt hypercortisolism served as a key differentiator from non−functional cystic lesions such as RCCs, as well as from the other differential diagnoses discussed above. A diagnostic misstep at this juncture could trigger an irreversible cascade of missed therapeutic opportunities. This case underscores the importance of a thorough evaluation to avoid such pitfalls and prevent inappropriate treatments that might accelerate disease progression.

Pituitary apoplexy requires personalized treatment strategies adapted to patient clinical characteristics (9). Therapeutic strategies targeting hormone-secretion lineages have been shown to improve prognostic outcomes in pituitary adenoma management. Pituitary microadenomas achieve surgical remission in 60%-90% of cases, with 5-year recurrence rates stabilizing at 5-10% in most cohorts, though patients under 25 years demonstrate significantly higher recurrence rates (10). Osteoporosis affects up to 40% of patients with Cushing’s disease, and fracture risk correlates strongly with hypercortisolism regardless of age (10). For pediatric patients, clinical management must prioritize both developmental outcomes and long-term disease control, necessitating integrated monitoring for recurrence and bone mineral density loss during follow-up.

While MRI aids in detecting sellar lesions, diagnosing ACTH-producing microadenomas remains challenging due to their subtle imaging features. These tumors are typically diminutive in size and remain radiographically occult in up to 50% of cases, even with advanced dynamic sequences (11). Even when a lesion is visible on MRI, tumor origin is misidentified in up to 30% of cases (6); suprasellar lesions, for example, are particularly prone to being initially misdiagnosed as intrasellar adenomas, sometimes leading to unnecessary surgical exploration (12). The absence of contrast enhancement in apoplexy can mimic Rathke’s cleft cysts or necrotic metastases, adding further diagnostic difficulty. Nevertheless, MRI remains the primary modality for initial evaluation, with histopathological analysis providing the definitive diagnosis.

## Conclusions

Pediatric ACTH-secreting pituitary apoplexy is a rare and diagnostically challenging entity. As this case illustrates, it can masquerade as a hypertensive emergency with acute renal impairment—a presentation not previously emphasized in the literature that necessitates heightened clinical vigilance and comprehensive diagnostic evaluation. Clinicians should consider pituitary pathology in any child presenting with malignant hypertension and neurological symptoms, even when imaging findings are atypical, as early surgical intervention and hormonal stabilization are paramount for recovery.

In children presenting with malignant hypertension and neurological symptoms–particularly when accompanied by Cushingoid features–pituitary apoplexy should be considered, even if imaging shows a non-enhancing cystic lesion mimicking Rathke’s cleft cyst or if the tumor appears to be a microadenoma. Early biochemical screening for ACTH-dependent hypercortisolism may aid in differential diagnosis and guide timely intervention.

## Data Availability

The original contributions presented in the study are included in the article/supplementary material. Further inquiries can be directed to the corresponding author.
